# Management of Systemic Cardiotoxicity Associated with Antidepressant Use in Patients with Depressive Disorders: A Systematic Review

**DOI:** 10.3390/jcm14113696

**Published:** 2025-05-25

**Authors:** Omar Anwar Saleh Al Nakhebi, Raluka Albu-Kalinovic, Adela Bosun, Oana Neda-Stepan, Marius Gliga, Cătălina-Angela Crișan, Ileana Marinescu, Virgil-Radu Enătescu

**Affiliations:** 1 Doctoral School, University of Medicine and Pharmacy Victor Babes Timisoara, 300041 Timisoara, Romania; raluka.kalinovic@umft.ro (R.A.-K.); adela.bosun@umft.ro (A.B.); oana.neda-stepan@umft.ro (O.N.-S.); marius.gliga@umft.ro (M.G.); 2 Discipline of Psychiatry and Pediatric Psychiatry, Department of Neurosciences, Iuliu Hatieganu University of Medicine and Pharmacy, 400347 Cluj-Napoca, Romania; ccrisan@umfcluj.ro; 3 Discipline of Psychiatry, Faculty of Medicine, University of Medicine and Pharmacy of Craiova, 200349 Craiova, Romania; ileana.marinescu@umfcv.ro; 4 Discipline of Psychiatry, Department of Neurosciences, University of Medicine and Pharmacy Victor Babes Timisoara, 300041 Timisoara, Romania; enatescu.virgil@umft.ro

**Keywords:** cardiotoxicity, antidepressive agents

## Abstract

**Background**: Depression is one of the leading causes of disability worldwide, with a significant impact on individuals’ quality of life. The use of antidepressants is fundamental in the treatment of this condition; however, it is essential to recognize that the use of these drugs can be associated with adverse effects, including cardiotoxicity. **Methods**: Using the Preferred Reporting Items for Systematic Reviews and Meta-Analyses (PRISMA) guidelines, a literature search was conducted of the PubMed/Medline, Scopus, and Cochrane databases. Twenty studies were included in the final quantitative analysis. The Joanna Briggs Institute critical appraisal tool was used to assess the risk of bias. **Results**: It is possible to highlight a correlation between intake of antidepressants and development of cardiotoxicity. Analysis of the selected studies allows for an understanding of the importance of a multidisciplinary approach for the protection of these patients. **Conclusions**: Management of antidepressant-induced cardiotoxicity requires a thorough understanding of the pathophysiological mechanisms involved and a critical evaluation of the various therapeutic strategies available to promote clinical practice.

## 1. Introduction

Depression represents a clinical condition with high prevalence, the therapeutic management of which frequently includes the administration of antidepressant drugs [1]. Although the association between depression and an increased risk of cardiovascular events is well-established, the influence of antidepressant use, with particular reference to selective serotonin reuptake inhibitors (SSRIs), on this risk still requires clarification and sparks lively debate in the scientific community [2].

Systemic cardiotoxicity refers to damage to and dysfunction of the heart caused by agents that circulate throughout the body. This type of toxicity can arise from various sources, including systemic cancer treatments such as chemotherapy and radiotherapy, but also from other drugs, environmental toxins, and substances of abuse. Systemic cardiotoxicity can manifest in several ways, including heart failure, arrhythmias, myocarditis, and cardiomyopathy [3]. Although effective in treating depression and anxiety disorders, antidepressant drugs are dangerous in case of overdose, with a high rate of hospitalization and mortality. Antidepressant poisoning mainly affects the nervous, cardiovascular, and endocrine systems, with cardiac arrhythmias being the leading cause of death [4]. Despite the introduction of new classes of antidepressant drugs, such as SSRIs and serotonin-norepinephrine reuptake inhibitors (SNRIs), tricyclic antidepressants (TCAs) remain a frequent cause of drug-related toxicity, with a high incidence of hospitalization and mortality [4,5]. Epidemiological studies show a hospitalization rate of 78.4% and a mortality rate of 0.73% [6].

Management of these intoxications requires a multidisciplinary approach, including ECG monitoring, the administration of activated charcoal and sodium bicarbonate, and, in severe cases of patients with refractory cardiogenic shock, pulmonary embolism, hypothermia, and cardiac arrest, the support of advanced resuscitation techniques such as veno-arterial extracorporeal membrane oxygenation (VA-ECMO) [7]. Furthermore, targeted temperature management (TTM) has proven effective in improving neurological outcomes and reducing mortality in patients who survive out-of-hospital cardiac arrest coma [8]. In addition to TCAs, other antidepressants such as bupropion, venlafaxine, quetiapine, and escitalopram can cause cardiovascular complications, including QT interval prolongation, arrhythmias, and cardiomyopathy [9]. Overdosing on these drugs can lead to serious consequences requiring immediate medical intervention.

Intravenous lipid emulsion (ILE) therapy is used in the treatment of toxicities related to lipophilic drugs, including some antidepressants. However, further studies are needed to define its role and specific indications [10].

Systemic cardiotoxicity is therefore a potential complication of antidepressant treatment, particularly with TCAs. This systematic literature review aims to analyze cases of antidepressant-induced cardiotoxicity secondary to the intake of antidepressant drugs, with the goal of understanding the optimal management and resolution of drug-related toxicity syndrome.

## 2. Materials and Methods

This systematic review was conducted according to the Preferred Reporting Items for Systematic Reviews and Meta-Analyses Statement (PRISMA) guidelines [11].

A systematic literature search was performed using the PubMed, Scopus, and Cochrane databases from January 2014 to March 2025 to retrieve only “case report”-type articles documenting the relationship between cardiotoxicity and antidepressant use in patients with depressive disorders.

The terms applied in the PubMed, Scopus, and Cochrane advanced search included “Antidepressant effect” and “Cardiotoxicity”. No studies were manually identified in the PubMed, Scopus, and Cochrane databases. The eligibility criteria were study type belonging to the case report category resulting from the search in the previously listed electronic databases that reported on the association between cardiotoxicity and antidepressant use in patients with depressive disorders. Articles had to be written in English and published between January 2014 and March 2025. The following exclusion criteria were applied: articles written in languages other than English, articles not belonging to the case report category (such as trials, systematic reviews, and meta-analyses), and studies conducted in species other than humans. Systematic reviews and meta-analyses were, however, read and consulted. Two independent researchers (O.A.S.A.N. and R.A.-K.) participated in this study, following a thorough research methodology that allowed for selecting articles, evaluating the relevance of the topic, and systematically analyzing abstracts before proceeding with a comprehensive evaluation of the full texts. Discrepancies were resolved by consulting a third supervising researcher (V.-R.E.), reaching an agreement rate of 0.98. In case of disagreement between the researchers, the study was not included in the following systematic review. 

After selection of the case reports, the following data were considered for each study and extracted using an Excel sheet: (a) author(s), (b) year of publication, (b) demographic characteristics, sample characteristics, (c) name of the antidepressant, (d) signs and symptoms, (e) treatment procedure used, and (f) post-operative conditions and follow-up. Two authors (O.A.S.A.N. and R.A.-K.) independently assessed the quality of the included studies using the “JBI Critical Appraisal Tools” [12], providing the same values. A third supervising researcher (V.-R.E.) was further consulted and agreed with the evaluations provided. The JBI Critical Appraisal Tools for evaluating the quality of quantitative studies are a checklist of eight questions. For each question on the checklist, one of the following judgments could be assigned: Yes/No/Unclear/Not Applicable (Table 1). The risk of bias, for each individual study selected, was classified as follows: high (H) if the study achieved a score with 49% “yes”, moderate (M) if the percentage of “yes” judgments was between 50% and 69%, and low (L) when the percentage of “yes” judgments was greater than 70%.

## 3. Results

### 3.1. Selection of the Studies

**Figure 1:**
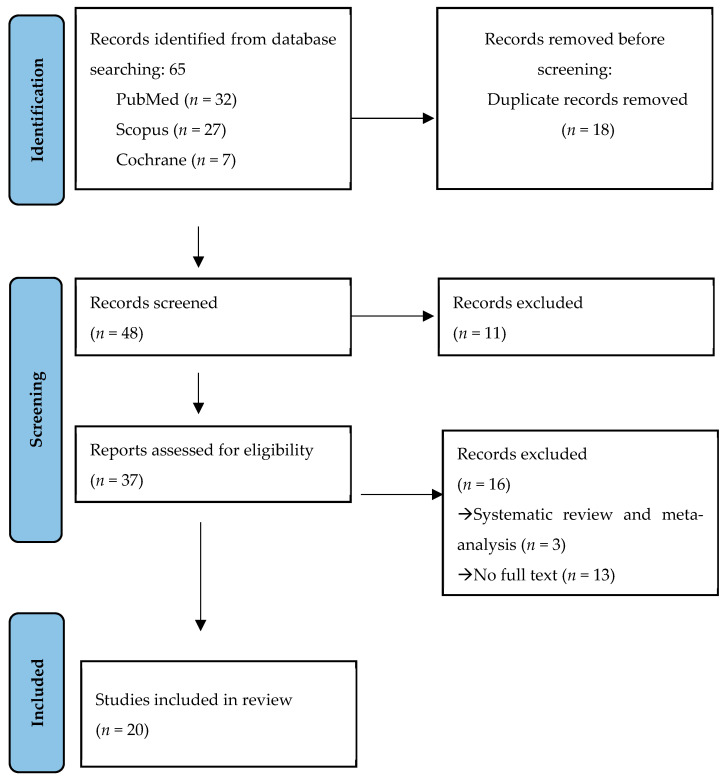
PRISMA flow diagram.

Initially, a search process was conducted that identified 65 records. After removing duplicates (*n* = 18), the remaining 48 studies were screened based on titles and abstracts, and 11 records were excluded. The remaining 37 studies were examined through a critical reading of the full text. Subsequently, 16 studies were excluded with reasons (Figure 1). In the end, a total of 20 studies [13,14,15,16,17,18,19,20,21,22,23,24,25,26,27,28,29,30,31,32] were included (Figure 1).

### 3.2. Results of the Studies

The following table (Table 2) shows the general characteristics of the included studies.

#### 3.2.1. Age and Gender Distribution of Patients

The age of the subjects ranged from 14 to 68 years, with an average age of 32.15 years. The male subjects (*n* = 7) had a mean age of 35.85 years, and the female subjects (*n* = 13) a mean age of 27.46 years.

#### 3.2.2. Antidepressant

Analysis of the 20 cases investigated the correlation between the use of antidepressant drugs and cases of cardiotoxicity. Nine patients had taken TCAs (seven amitriptyline and two imipramine), five patients had taken bupropion, five SSRIs and SNRIs (four venlafaxine and one escitalopram), one aripiprazole, and one mirtazapine.

#### 3.2.3. Signs and Symptoms

The typical symptoms of severe cardiovascular toxicities including arrhythmia characterized by QT elongation, cardiac arrest, cardiogenic shock, coma, and respiratory distress.

#### 3.2.4. Treatment and Post-Operative Conditions

Treatment for antidepressant cardiotoxicity focuses on stabilizing vital functions and treating cardiac complications. Sodium bicarbonate is often used to treat QRS complex widening and ventricular arrhythmias. In some cases, advanced support therapies, such as ECMO (extracorporeal membrane oxygenation) and TTM (targeted temperature management), may be necessary.

#### 3.2.5. Follow-Up

Not all patients were followed up with after discharge from the intensive care unit.

#### 3.2.6. Risk of Bias

Of the 20 articles included in the review, most have a low risk of bias (*n* = 17). The remaining three are at moderate risk (Table 3).

## 4. Discussion

Depression represents a common clinical condition that frequently occurs in comorbidity with cardiovascular diseases; a significant prevalence is found in patients with heart disease [33]. Antidepressants are a widely prescribed class of psychotropic drugs for the treatment of a broad spectrum of psychiatric disorders. Although their primary indication is for major depressive disorder, their use extends to anxiety disorders, obsessive-compulsive disorder, panic disorder, post-traumatic stress disorder, and other related conditions [33,34]. The primary goal of antidepressant therapy is to modulate dysfunctional emotional states, such as anxiety, profound sadness, or anger, bringing them back within a physiological range that allows the patient to regain a satisfactory quality of life, which is often significantly compromised by psychological or emotional distress [33,34].

Despite documented therapeutic benefits, the use of antidepressants is associated with a wide spectrum of potential risks and side effects that can range from mild and transient disturbances to severe adverse reactions [35]. Therefore, a rigorous and individualized assessment of the risk–benefit ratio is an indispensable prerequisite before starting, and during, any antidepressant treatment. The choice of the most appropriate drug for a given patient cannot disregard careful consideration of its expected efficacy for the specific condition being treated, its tolerability profile, the patient’s clinical and medical history, and potential drug interactions [33,34,35].

The literature has shown that SSRIs and some SNRIs are generally better tolerated than TCAs and MAOIs, which is why they represent the first-line treatment for depression and anxiety [36]. More serious complications require careful clinical monitoring and, in some cases, timely medical intervention. Among these conditions is serotonin syndrome, which typically manifests with a symptomatic triad that includes altered mental status, hyperactivity of the autonomic nervous system, and neuromuscular abnormalities [36]. The risk of developing this syndrome increases significantly in case of association between SSRIs/SNRIs and MAOIs, which is absolutely contraindicated, but the danger also exists with the co-administration of triptans, tramadol, lithium, linezolid, fentanyl, or St. John’s wort [36]. The severity of serotonin syndrome requires immediate discontinuation of all suspected serotonergic agents and implementation of intensive supportive measures, which may include hyperthermia control, sedation, and, in the most severe cases, admission to intensive care [26,36].

A further concern associated with the use of antidepressants is the potential increased risk of suicidal ideation and behavior, particularly in children, adolescents, and young adults during the first few weeks of treatment or following dosage changes [37].

Although rare, hepatotoxicity is a potential risk associated with several antidepressants, with reports of variable liver damage for MAOIs, nefazodone, bupropion, duloxetine, trazodone, and agomelatine [38]. Several antidepressants, especially SSRIs and SNRIs, can also interfere with platelet function, increasing the bleeding risk, which can manifest as ecchymosis, epistaxis, and gastrointestinal or gynecological bleeding [39]. The administration of MAOIs can also be associated with severe and specific hypertensive crisis [40]. All antidepressants have the potential to lower the seizure threshold and induce epileptic seizures, especially at high dosages or in predisposed patients. Bupropion is associated with a relatively higher risk and is contraindicated in patients with seizure disorders or risk factors [24].

Hyponatremia represents a relevant side effect associated with the use of SSRIs and SNRIs, particularly in the elderly and in those taking diuretics, manifesting with non-specific symptoms, and in severe forms, seizures, coma, and death can occur [40]. Cardiotoxicity is an significant risk primarily associated with TCAs. However, it can also occur with other classes of antidepressants, albeit to a less extent or through distinct mechanisms [34]. TCAs are known for their ability to induce cardiac arrhythmias, sinus tachycardia, orthostatic hypotension, and alterations in cardiac conduction, with a risk of rapidly fatal outcome in case of overdose. For these reasons, their use is generally contraindicated or requires extreme caution in patients with pre-existing significant heart disease or rhythm or conduction disorders [2,3]. SSRIs/SNRIs generally have a more favorable cardiovascular safety profile; however, citalopram and escitalopram can cause dose-dependent prolongation of the QT interval, increasing the risk of serious ventricular arrhythmias, especially in the presence of pre-existing risk factors or at high dosages [19].

The problems described above are often interconnected, highlighting the need for a holistic clinical evaluation and careful and proactive patient monitoring. It is therefore essential that any therapeutic treatment with antidepressants is personalized, considering the patient’s characteristics, comorbidities, and side effects. The assessment of tolerability must consider both rare and severe events and the impact of common effects on well-being and therapy continuity [34,37].

The cardiovascular safety of antidepressants is a primary concern, especially in those patients who already have a vulnerability due to the depression itself or the presence of pre-existing cardiac conditions [34]. This study focused on analysis of clinical cases that allowed evaluation of the multidisciplinary management of cardiotoxicity associated with the use of antidepressants in patients with depressive disorders. The aim was to evaluate the effectiveness of an integrated approach involving cardiologists, psychiatrists, and general practitioners in reducing the incidence and severity of adverse events.

The incidence of cardiotoxicity in patients taking antidepressants, as highlighted in the literature by Nguyen et al. and Ikejiri et al., increases when these individuals take TCAs [13,14]. In these cases, typical manifestations of cardiotoxicity include prolongation of the QT interval, sinus tachycardia, and the presence of prolonged QRS complexes [13,15,17]. In some cases, ventricular arrhythmias, such as ventricular tachycardia and ventricular fibrillation, can occur [13,14]. Symptoms associated with antidepressant-induced cardiotoxicity can vary from patient to patient and may include palpitations, chest pain, syncope, dyspnea, peripheral edema, and seizures [13,14,15]. Timely management of these complications is crucial for patient survival [16,17]. Cardiac monitoring via ECG and echocardiogram, as well as blood pressure monitoring, are crucial in the early identification of signs of cardiotoxicity, thus allowing for timely intervention (drugs such as bupropion, venlafaxine, quetiapine, and escitalopram can indeed be responsible for QT interval prolongation, arrhythmias, and cardiomyopathy [18,20,22,23,24]).

Several therapeutic strategies can be employed in the management of cardiotoxicity induced by antidepressant drugs; a multidisciplinary approach is fundamental in these cases. Collaboration among cardiologists, psychiatrists, and general practitioners has allowed for optimization of pharmacological treatment, close patient monitoring, and timely intervention in the case of adverse cardiac events. In particular, strategies such as dosage or type of antidepressant modification, drug discontinuation, and specific pharmacological treatment for cardiotoxicity have significantly contributed to reducing the incidence and severity of cardiac complications [13,14,15]. Treatment of cardiotoxicity associated with antidepressants focuses on stabilization of vital functions, gastrointestinal decontamination, and treatment of cardiac complications. Sodium bicarbonate is often used to treat QRS complex widening and ventricular arrhythmias [16,21]. In some cases, advanced supportive therapies, such as ECMO and TTM, may be necessary to stabilize the patient and ensure survival [14,15].

The administration of sodium bicarbonate represents a fundamental therapeutic strategy, especially in cases of cardiotoxicity induced by TCAs. The scientific evidence for the effectiveness of sodium bicarbonate in the treatment of TCA cardiotoxicity is consistent; numerous studies and case reports demonstrate its efficacy in treating QRS interval prolongation and ventricular arrhythmias induced by TCAs [41,42]. In some rare cases of severe refractory cardiotoxicity, the administration of high doses of bicarbonate has led to resolution of the clinical picture [43]. Studies conducted on animal models have also supported the efficacy of bicarbonate in reducing amitriptyline-induced cardiotoxicity. Clinical indications for the use of sodium bicarbonate in antidepressant cardiotoxicity include QRS interval prolongation greater than 100 ms, the presence of ventricular arrhythmias, and hypotension refractory to fluid administration, as well as toxin-induced metabolic acidosis [44,45]. However, sodium bicarbonate therapy is not without limitations and adverse effects. Excessive use can lead to alkalosis, hypernatremia, hypokalemia, and hypocalcemia, and excessive doses can even be fatal [44]. Furthermore, sodium bicarbonate may be ineffective in treating cardiotoxicity induced by some drugs that block intercellular junctions, such as bupropion. Recommended dosage protocols include an initial intravenous bolus of 1–2 mEq/kg, repeated every 3–5 min until improvement of the ECG tracing, possibly followed by a continuous infusion of 150 mEq in 1 L of 5% dextrose solution at a rate of 150–200 mL/h to maintain arterial pH between 7.5 and 7.55 [16,17,43]. During therapy, continuous monitoring of ECG, arterial pH, and electrolytes is essential, with the goal of achieving normalization of the QRS or a value below 140 ms, a pH between 7.5 and 7.55, and a maximum sodium level of 155 mmol/L [43,44,45,46].

In some cases of severe cardiotoxicity induced by antidepressants, standard therapies may not be sufficient to stabilize the patient, and advanced supportive therapies, such as ECMO and TTM, are used to ensure survival and reduce the risk of complications [22]. ECMO is a technique that allows for support of the patient’s cardiac and respiratory function, allowing the heart and lungs to rest and recover. It is used in cases of severe cardiac and/or respiratory failure when the organs are unable to perform their function adequately [14]. In the context of antidepressant cardiotoxicity, ECMO may be indicated in the presence of cardiac arrest refractory to cardiopulmonary resuscitation; cardiogenic shock refractory to pharmacological treatment; malignant ventricular arrhythmias resistant to antiarrhythmic therapy; and severe acute respiratory failure.

The main indications for the use of VA-ECMO in this context include refractory cardiogenic shock and refractory cardiac arrest, as well as hemodynamic instability associated with ventricular arrhythmias [14]. VA-ECMO can also be used as a “bridge” to recovery of cardiac function [14]. Evidence of the effectiveness of VA-ECMO in this context demonstrates a significant improvement in hemodynamic and metabolic parameters in treated patients, with increased survival in patients with drug-induced cardiogenic shock [47]. Case reports have documented success in the treatment of toxicity from TCAs, bupropion, and venlafaxine using VA-ECMO [31].

Regarding timing and selection criteria, early implementation of VA-ECMO appears to be crucial for improving outcomes [47]. VA-ECMO is indicated in cases of refractory shock and cardiac arrest despite conventional therapies. However, adequate patient selection is essential to maximize benefits and minimize risks [48,49]. The benefits of VA-ECMO include immediate hemodynamic and respiratory support, the possibility of gaining time for drug metabolism and elimination, and the possibility of administering antidotes, if available. Risks associated with VA-ECMO include bleeding, thrombosis, limb ischemia, infections, and neurological complications [48]. Therefore, it is essential to carefully balance the potential benefits with the risks associated with this therapy [14,22,31].

TTM is a technique that involves lowering the patient’s body temperature to reduce neurological damage after cardiac arrest [14]. Therapeutic hypothermia slows down brain metabolism, reducing oxygen demand and limiting cell death. In the context of antidepressant cardiotoxicity, TTM may be indicated in the presence of cardiac arrest with return of spontaneous circulation (ROSC) but with severe neurological damage; prolonged seizures or status epilepticus; and malignant hyperthermia. TTM is generally maintained for 24–48 h, after which body temperature is gradually returned to normal values [14,15].

A reduction in mortality and an improvement in neurological outcomes have been demonstrated in patients undergoing TTM after cardiac arrest [14]. In particular, TTM has proven effective in cases of TCA-induced cardiac arrest, often in combination with VA-ECMO. It is important to consider that TTM can potentially interact with antidepressants by affecting the cardiac conduction system, causing prolongation of QT and QRS intervals on the ECG. Furthermore, TTM can decrease the activity of cytochrome CYP2D6, an enzyme involved in the metabolism of some antidepressants such as amitriptyline and nortriptyline. Therefore, these potential drug interactions must be carefully considered. Recommended TTM protocols generally involve maintaining a target temperature between 32 °C and 36 °C for at least 24 h, followed by slow and controlled rewarming to avoid intracranial pressure spikes. Continuous monitoring of body temperature and appropriate management of shivering, which can interfere with achieving and maintaining the target temperature, are essential throughout the process [14,15].

The use of ECMO and TTM requires careful evaluation of the risk–benefit ratio, as these are invasive techniques that can lead to complications. The decision to use these therapies should be made by a multidisciplinary team including cardiologists, intensivists, and other specialists, based on the patient’s clinical condition and available resources. It is important to emphasize that ECMO and TTM are supportive therapies that do not treat the underlying cause of cardiotoxicity. Therefore, it is essential to address the drug toxicity and manage the complications associated with overdose.

It should also be emphasized, as highlighted by Maina et al., that treatment-resistant depression (TRD) represents a significant clinical challenge today, characterized by failure to respond to adequate doses of at least two different classes of antidepressants [50]. In these patients, pharmacological management can become complex and increase the risk of secondary toxicity. Pharmacological options for TRD include different classes of antidepressants, but also non-pharmacological treatments such as neurostimulation and psychotherapies, usable in various strategies of augmentation, switching, polytherapy (combination of multiple antidepressants or the addition of augmenting drugs such as lithium or atypical antipsychotics), and the use of more recent drugs with different mechanisms of action (e.g., agomelatine, vortioxetine, esketamine). In this context, esketamine represents a therapeutic option with a manageable safety profile, but its efficacy and positioning in clinical practice require careful evaluation, especially in relation to the risk of polytherapy toxicity [50].

It is crucial to highlight how prevention represents a critical aspect in the management of antidepressant poisoning. Particularly in low- and middle-income countries, where access to mental health resources is limited and inadequate drug regulation can contribute to inappropriate use of TCAs, the implementation of education and training strategies for healthcare professionals and the general population is essential.

Although the objective of this systematic review has been achieved, it should be emphasized that the analysis of case reports alone within the considered time range may represent a limitation of the study, and therefore it would be useful to implement further types of studies such as case-control studies, cohort studies, and randomized controlled trials, increasing the sample under examination.

## 5. Conclusions

Cardiotoxicity induced by antidepressant drugs represents a serious complication that can arise in patients with depression, with a significant impact on morbidity and mortality. Management of this condition requires a thorough understanding of the pathophysiological mechanisms involved and a critical evaluation of the various therapeutic strategies available. Each therapeutic strategy has specific indications and limitations that must be carefully considered in clinical practice. Despite the current evidence, further research is needed to optimize treatment protocols for antidepressant-induced cardiotoxicity. In particular, randomized controlled trials are needed to evaluate the efficacy of VA-ECMO and TTM in this specific context and to define optimal criteria for their application. Further research should also focus on optimizing sodium bicarbonate dosage protocols and identifying innovative therapeutic strategies for the management of cardiotoxicity induced by different classes of antidepressants.

The use of a multidisciplinary approach in the management of patients with antidepressant cardiotoxicity, involving cardiologists, psychiatrists, intensivists, and clinical pharmacologists, is of fundamental importance to ensure optimal and personalized treatment. Future research should also explore the early identification of patients at risk of developing cardiotoxicity by implementing the introduction of targeted preventive strategies.

## Figures and Tables

**Table 1:** Checklist for the quality system tool.

Checklist
1. Were the Patient’s Demographic Characteristics Clearly Described? Yes/No/Unclear/Not Applicable
2. Was the Patient’s History Clearly Described and Presented as a Timeline? Yes/No/Unclear/Not Applicable
3. Was the Current Clinical Condition of the Patient on Presentation Clearly Described? Yes/No/Unclear/Not Applicable
4. Were Diagnostic Tests or Assessment Methods and the Results Clearly Described? Yes/No/Unclear/Not Applicable
5. Was the Intervention(s) or Treatment Procedure(s) Clearly Described? Yes/No/Unclear/Not Applicable
6. Was the Postintervention Clinical Condition Clearly Described? Yes/No/Unclear/Not Applicable
7. Were Adverse Events (Harms) or Unanticipated Events Identified and Described? Yes/No/Unclear/Not Applicable
8. Does the Case Report Provide Takeaway Lessons? Yes/No/Unclear/Not Applicable

**Table 2:** General characteristics of the included studies.

S. No	First Author’s Name/Year of Publication [Reference Number]	Age and Sex of Patient	Antidepressant	Signs and Symptoms	Treatment and Post-Operative Conditions	Follow Up
1	Nguyen H/2021 [13]	35-year-old female	Tricyclic antidepressant (amitriptyline)	Severe cardiovascular toxicities with arrhythmia characterized by QT elongation.	The patient was admitted to the intensive care unit, where hemodynamic parameters and ECG were monitored; she regained consciousness after 2 days.	After 7 days the patient did not reveal any recurrence.
2	Ikejiri K/2021 [14]	19-year-old male	Tricyclic antidepressant (amitriptyline)	The patient presented with altered mental status and a seizure. The following values were assessed: initial body temperature (36.1 °C), heart rate (86 bpm), blood pressure (92/39 mm Hg), and SpO2 (98%). Upon admission, the patient exhibited a comatose state with a Glasgow Coma Scale score of 3 (E1V1M1) with a generalized tonic-clonic seizure. His urine test result was positive for Amitriptyline.	The patient was admitted and intubated. The ECG showed a wide QRS complex tachycardia. Following ingestion, the patient experienced a TCA-induced cardiac arrest. The patient was initiated on VA-ECMO 240 min later, which stabilized the hemodynamic status, and the ECG gradually improved. The patient was weaned off ECMO after 27 h. After completing targeted temperature management, their mental status improved, and they were extubated on day 5.	He presented with no neurological deficits and was discharged on day 15.
3	Zitoune Z/2023 [15]	55-year-old female	Tricyclic antidepressant (imipramine)	The patient was found unconscious on site and presented with arrhythmia, cardiac arrest, cardiogenic shock, coma, and respiratory distress.	The patient presented with severe rhabdomyolysis and rapidly developed cardiogenic shock and malignant arrhythmias requiring VA-ECMO. Continuous renal replacement therapy was initiated after admission. Serial blood measurements of imipramine and its active metabolite desipramine were performed. Cardiac function improved, and ECMO was explanted after 4 days. The severe acute respiratory distress syndrome resolved spontaneously, and the neurological outcome was favorable despite early myoclonus. The patient regained consciousness on the fifth day.	The patient was discharged after 4 weeks.
4	Amiri H/2016 [16]	27-year-old male	Clonazepam and tricyclic antidepressant (amitriptyline)	The patient presented with status epilepticus, hypotension, and refractory QRS complex widening.	The patient was admitted to intensive care unit, where he resolved after intravenous administration of 2650 mEq sodium bicarbonate.	He was discharged after 1 week with no symptoms.
5	Kassim T/2018 [17]	21-year-old male	Tricyclic antidepressant (amitriptyline)	The patient presented unconscious after an amitriptyline overdose as a suicide attempt.	The patient was admitted to the intensive care unit. The ECG showed sinus tachycardia. Intravenous fluids and sodium bicarbonate were administered. The patient was taken off mechanical ventilation after 2 days with signs of improvement.	The patient was discharged after 7 days of hospitalization. At the 1-month follow-up, the troponin level was repeated and was within normal limits.
6	Robinson S/2022 [18]	14-year-old female	Bupropion	The patient ingested 15 g of bupropion, resulting in the onset of status epilepticus, with QT prolongation evolving into ventricular tachycardia and ventricular fibrillation, requiring five cardioversions and one defibrillation.	The QT interval eventually narrowed after supportive care and lidocaine drip. The patient was able to be extubated two days later, with cognitive function and good echocardiogram.	-
7	Koshy P/2023 [19]	22-year-old female	Selective serotonin reuptake inhibitor (escitalopram)	The patient presented with the following values: heart rate 102 bpm, blood pressure 130/80 mmHg, respiratory rate 20 rpm, oxygen saturation 97% on room air, and temperature 36.4 °C. She was fully conscious and oriented.	Her ECG showed T-wave inversions that resolved the next day with supportive management. After 24 h, she developed dystonia, which resolved with low doses of benzodiazepines.	She became stable and was discharged on the fifth day after admission.
8	Cobilinschi C/2023 [20]	33-year-old male	Selective serotonin and norepinephrine reuptake inhibitor (venlafaxine)	The patient was cardiorespiratory stable but unresponsive with a GCS of 4, was intubated, and mechanical ventilation was initiated.	After admission to the intensive care unit, he developed cardiovascular collapse refractory to vasopressors with junctional bradycardia, followed by spontaneous conversion to sinus rhythm. This was followed by ventricular extrasystoles, trigeminy, and even episodes of non-sustained ventricular tachycardia. Generalized tonic-clonic seizures were observed. Antiarrhythmic and anticonvulsant therapy, an intravenous lipid emulsion bolus, and continuous infusion were then administered. His condition progressively improved over the following hours, and 24 h later the vasopressor was gradually discontinued. On day 2, the patient had a recurrence of cardiovascular collapse, and a second dose of intravenous lipid emulsion was administered.	The patient was discharged in good condition on day 15 and referred to a psychiatrist.
9	Ando M/2020 [21]	68-year-old male	Tricyclic antidepressant (amitriptyline)	The patient was found with impaired awareness. Upon admission, the GCS score was 3 (E1; V1; M1); systolic/diastolic blood pressure, 101/62 mm Hg; oxygen saturation, 94%; body temperature, 38.7 °C; heart rate, 120/min; respiration rate, 20/min; blood pH, 7.022; QTc interval, 610 ms; and QRS interval, 270 ms.	Atrial fibrillation was evident on the electrocardiogram. The patient was subsequently intubated and treated for a shock-like hemodynamic status. The patient’s level of consciousness improved 60 h after admission. On the 10th day, he was transferred to the medical psychiatry unit.	In the unit, the patient reported transient suicidal feelings that gradually dissipated. On the 23rd day, he was discharged.
10	Reinsch N/2021 [22]	18-year-old female	Bupropion	Bupropion ingestion triggered status epilepticus, prolonged QTc, widened QRS, pulseless ventricular tachycardia, and cardiovascular collapse necessitating ECMO and Impella support.	This patient exhibited a widening QRS complex despite aggressive bicarbonate therapy and recurrent episodes of pulseless ventricular tachycardia, which were ultimately resolved with lidocaine. Neurological prognosis was complicated by the absence of brainstem reflexes. Following therapy, the patient was weaned off Impella, ECMO, and the ventilator after the seventh day of hospitalization.	She was discharged on hospital day 17 with a plan for intensive outpatient psychiatric therapy.
11	Angel-Isaza AM/2020 [23]	28-year-old female	Tricyclic antidepressant (amitriptyline)	The patient developed cardiac arrest and received advanced cardiopulmonary resuscitation.	Hypotension and pulselessness did not respond to sodium treatment. The patient stabilized following treatment with lipid emulsion therapy and was weaned off vasopressors and mechanical ventilation over the next 24 h without residual neurological deficits.	-
12	Ungureanu R/2025 [24]	25-year-old male	Bupropion	The patient was admitted to a psychiatric clinic for a suicide attempt by self-poisoning with bupropion; shortly thereafter, she was transferred to the hospital for rhabdomyolysis and hepatic cytolysis syndrome. No abnormalities were observed during the physical examination.	The patient presented with a bupropion overdose. The absence of typical neurotoxic or muscular symptoms and the subsequent involvement of multiple factors led to a decision to initiate early renal replacement therapy, despite the absence of overt acute kidney injury.	On day 4, the patient was discharged in stable condition and referred to a mental health center.
13	Franco V/2015 [25]	30-year-old female	Bupropion	The patient presented to the emergency department with four seizure episodes following the ingestion of extended-release bupropion. The patient’s vital signs upon arrival were blood pressure 97/45 mm Hg, heart rate 102 beats/minute, respiratory rate 19 breaths/minute, and O2 saturation 97%.	The patient was intubated and sedated. Subsequently, she was treated with sodium bicarbonate for tachycardia with QT interval prolongation.	-
14	Garcia S/2017 [26]	58-year-old male	Selective serotonin and norepinephrine reuptake inhibitor (venlafaxine)	The patient reported experiencing intermittent palpitations associated with exertional dyspnea and added that he had suffered from asthenia and a lack of endurance since early childhood; however, his fatigue symptoms worsened when he started taking venlafaxine approximately twelve years ago.	Electrocardiogram (ECG) examination revealed symptoms of skipped beats and a racing heart, associated with sinus rhythm and occasional premature atrial beats. The patient underwent a dobutamine stress test. His ejection fraction response increased from 60% at rest to 80% at peak stress. The patient’s genetic test results established the phenotype of a CYP2D6 poor metabolizer and a CYP2C19 intermediate metabolizer.	The recommendations included switching the patient to alternative agents. Desvenlafaxine was recommended.
15	Avcil M/2016 [27]	30-year-old female	Aripiprazole	She arrived at the emergency department in critical condition approximately 2 h after drug ingestion. Upon arrival, she exhibited signs of cardiotoxicity, including QT interval prolongation and atrial fibrillation, in addition to profound hypotension and severe depression of the central nervous and respiratory systems.	The patient was admitted to the emergency critical care unit, where she was treated with a sodium bicarbonate infusion (20 mEq/h) for acidosis found in the blood gas analysis, and potassium replacement was also started. Because the patient exhibited persistent hypotension, and her metabolic condition did not improve, intravenous lipid emulsion (ILE) therapy was administered. Following this procedure, the ECG returned to sinus rhythm, and the QT interval normalized.	Her overall condition improved after extubation, no further issues were encountered, and she was discharged from the hospital 4 days after admission. Her QTc at discharge was 425 ms. During a follow-up appointment 20 days later, the patient’s only complaint was mild hoarseness.
16	Castanares-Zapatero D/2016 [28]	45-year-old female	Selective serotonin and norepinephrine reuptake inhibitor (venlafaxine)	Upon arrival at the emergency facilities, the patient was found to be drowsy, with a Glasgow Coma Scale (GCS) score of 13/15, and presented with the following vital signs: arterial blood pressure 145/84 mmHg, heart rate 100 min^−1^. The neurological examination showed no particularities, with the exception of horizontal nystagmus. The electrocardiogram revealed a sinus rhythm at 100 min^−1^, with narrow QRS complexes and a QTc duration of 430 ms.	Due to the progression of altered consciousness, the patient was transferred to the intensive care unit. The venlafaxine overdose resulted in marked left ventricular dysfunction, in the absence of significant conduction disorders on the electrocardiogram.	The patient left the ICU on day 3, without having manifested any symptoms associated with serotonin syndrome. Complete left ventricular function recovery was observed on echocardiogaphy at the 2-week follow-up.
17	Kontio/2015 [29]	19-year-old female	Tricyclic antidepressant (amitriptyline)	The cardiac arrest was witnessed, but no bystander cardiopulmonary resuscitation (CPR) was performed. Three defibrillations, magnesium sulfate, and sodium bicarbonate were given and her trachea was intubated, after which return of spontaneous circulation (ROSC) was achieved in 26 min. After ROSC, she had seizures and was sedated.	The patient was unconscious and had dilated pupils. She was tachycardic with a body temperature of 33.5 °C. She was transferred to the intensive care unit was maintained with invasive cooling. During the treatment, she did not experience any serious cardiac arrhythmia, transthoracic echocardiogram was normal, and the electrocardiogram (ECG) returned to normal. The patient was extubated 45 h after the cardiac arrest. After the extubation, she was alert and cooperative, but slightly delusional.	She was transferred to a ward on the third day and discharged from hospital on the sixth day of admission. Ambulatory psychiatric follow-up was organized.
18	Azdaki N/2019 [30]	19-year-old female	Tricyclic antidepressant (imipramine)	The patient presented to our emergency department with primary complaints of heart palpitations, weakness, and lethargy. She reportedly had experienced two to three episodes of palpitations within the 2 weeks prior to presentation. Upon admission to the emergency department, the patient became unconscious and experienced a drop attack. She immediately underwent cardiopulmonary monitoring, and an electrocardiogram (ECG) was performed, which showed regular wide complex tachycardia.	The patient was diagnosed with PSVT. A drug screening urine test was performed. She had ingested imipramine. Sodium bicarbonate (15 cc twice) and calcium gluconate were administered. Despite the administration of amiodarone, the patient still had arrhythmia and, given the patient’s better reaction to lidocaine, amiodarone was substituted with lidocaine. Infusion of lidocaine and sodium bicarbonate and gastric decontamination were continued in the ICU, and the QRS complex narrowed on the ECG.	-
19	Matsumoto H/2015 [31]	24-year-old female	Mirtazapine	The patient with depression was found in a comatose state, and an emergency life-saving technology service was called. After transport to the hospital, she stopped breathing, became pulseless, and immediate cardiopulmonary life support was initiated. Electrocardiographic monitoring showed asystole during resuscitation even after arrival at the hospital.	In addition to the conventional administration of adrenaline (total dose, 5 mg), 20% intralipid 100 mL was administered intravenously 8 min after arrival at the hospital and administered again 27 min after re-arrival at the hospital due to persistent asystole. Return of spontaneous circulation occurred 29 min after arrival (70 min after cardiac arrest).	The patient recovered without major complications and was transferred to another hospital for psychiatric care 70 days after admission.
20	Galust H/2023 [32]	53-year-old female	Selective serotonin and norepinephrine reuptake inhibitor (venlafaxine)	The electrocardiogram showed sinus tachycardia with an enlarged QRS complex but normal corrected QT interval. The patient was rapidly intubated and mechanically ventilated. She received fluids and 8.4% sodium bicarbonate. Norepinephrine infusion was started.	Initial management of cardiovascular failure included dobutamine, norepinephrine, and epinephrine infusions to maintain blood pressure. Due to refractoriness of the presumed drug-induced cardiac failure with rapidly worsening multiorgan failure, veno-arterial ECMO was implemented via femoral vessel cannulation at the bedside.	The ICU stay was complicated by the onset of ventilator-associated pneumonia related to *Enterobacter cloacae*, treated with cefepime, and by several hemorrhage episodes at the cannulation site requiring red blood cell, platelet, and fresh plasma transfusions. The patient was discharged to the medical ward after 25 days of hospitalization.

**Table 3:** Risk of bias evaluation of the included studies, using the Joanna Briggs Institute critical appraisal checklist for case report (L = Low, M = Moderate, H = High).

Sl. No	First Author’s Name/Year of Publication	1. Were the Patient’s Demographic Characteristics Clearly Described?	2. Was the Patient’s History Clearly Described and Presented as a Timeline?	3. Was the Current Clinical Condition of the Patient on Presentation Clearly Described?	4. Were the Diagnostic Tests or Assessment Methods and the Results Clearly Described?	5. Was the Intervention(s) or Treatment Procedure(s) Clearly Described?	6. Was the Postintervention Clinical Condition Clearly Described?	7. Were Adverse Events (Harms) or Unanticipated Events Identified and Described?	8. Does the Case Report Provide Takeaway Lessons?	Overall Risk of Bias
1	Nguyen H/2021 [13]	Yes	Yes	Yes	Yes	Yes	Yes	No	Yes	L
2	Ikejiri K/2021 [14]	Yes	Yes	Yes	Yes	Yes	Yes	Yes	Yes	L
3	Zitoune Z/2023 [15]	Yes	Yes	Yes	Yes	Yes	Yes	Yes	Yes	L
4	Amiri H/2016 [16]	Yes	Yes	Yes	Yes	Yes	Yes	No	Yes	L
5	Kassim T/2018 [17]	Yes	Yes	Yes	Yes	Yes	Yes	No	Yes	L
6	Robinson S/2022 [18]	Yes	Yes	Yes	Yes	Yes	No	No	Yes	M
7	Koshy P/2023 [19]	Yes	Yes	Yes	Yes	Yes	Yes	Yes	Yes	L
8	Cobilinschi C/2023 [20]	Yes	Yes	Yes	Yes	Yes	Yes	Yes	Yes	L
9	Ando M/2020 [21]	Yes	Yes	Yes	Yes	Yes	Yes	Yes	Yes	L
10	Reinsch N/2021 [22]	Yes	Yes	Yes	Yes	Yes	Yes	Yes	Yes	L
11	Angel-Isaza AM/2020 [23]	Yes	Yes	Yes	Yes	Yes	No	No	Yes	M
12	Ungureanu R/2025 [24]	Yes	Yes	Yes	Yes	Yes	Yes	No	Yes	L
13	Franco V/2015 [25]	Yes	Yes	Yes	No	Yes	No	No	Yes	M
14	Garcia S/2017 [26]	Yes	Yes	Yes	Yes	Yes	No	No	Yes	L
15	Mucahit A/2016 [27]	Yes	Yes	Yes	Yes	Yes	Yes	Yes	Yes	L
16	Castanares-Zapatero D/2016 [28]	Yes	Yes	Yes	Yes	Yes	Yes	Yes	Yes	L
17	Kontio/2015 [29]	Yes	Yes	Yes	Yes	Yes	Yes	Yes	Yes	L
18	Azdaki N/2019 [30]	Yes	Yes	Yes	Yes	Yes	Yes	No	Yes	L
19	Matsumoto H/2015 [31]	Yes	Yes	Yes	Yes	Yes	Yes	Yes	Yes	L
20	Galust H/2023 [32]	Yes	Yes	Yes	Yes	Yes	Yes	Yes	Yes	L
